# RNA Binding Motif Protein RBM45 Regulates Expression of the 11-Kilodalton Protein of Parvovirus B19 through Binding to Novel Intron Splicing Enhancers

**DOI:** 10.1128/mBio.00192-20

**Published:** 2020-03-10

**Authors:** Jianke Wang, Safder S. Ganaie, Fang Cheng, Peng Xu, Kang Ning, Xiaomei Wang, Steve Kleiboeker, Shipeng Cheng, Jianming Qiu

**Affiliations:** a Institute of Special Animal and Plant Sciences, Chinese Academy of Agricultural Sciences, Changchun, Jilin, China; b Department of Microbiology, Molecular Genetics and Immunology, University of Kansas Medical Center, Kansas City, Kansas, USA; c Department of Research and Development, Viracor Eurofins Laboratories, Lee’s Summit, Missouri, USA; Virginia Polytechnic Institute and State University

**Keywords:** RBM45, mRNA splicing, intron splicing enhancer, parvovirus B19, 11-kDa protein, RNA binding proteins, RNA processing, parvovirus

## Abstract

During infection of human parvovirus B19 (B19V), one viral precursor mRNA (pre-mRNA) is transcribed by a single promoter and is alternatively spliced and alternatively polyadenylated. Here, we identified a novel *cis*-acting sequence (5′-GUA AAG CUA CGG GAC GGU-3′), intronic splicing enhancer 3 (ISE3), which lies 72 nucleotides upstream of the second splice acceptor (A2-2) site of the second intron that defines the exon of the mRNA encoding the 11-kDa viral nonstructural protein. RNA binding motif protein 45 (RBM45) specifically binds to ISE3 with high affinity (equilibrium dissociation constant [*K_D_*] = 33 nM) mediated by its RNA recognition domain and 2-homo-oligomer assembly domain (RRM2-HOA). Knockdown of RBM45 expression or ectopic overexpression of RRM2-HOA in human erythroid progenitor cells (EPCs) expanded *ex vivo* significantly decreased the level of viral mRNA spliced at the A2-2 acceptor but not that of the mRNA spliced at A2-1 that encodes VP2. Moreover, silent mutations of ISE3 in an infectious DNA of B19V significantly reduced 11-kDa expression. Notably, RBM45 also specifically interacts *in vitro* with ISE2, which shares the octanucleotide (GGGACGGU) with ISE3. Taken together, our results suggest that RBM45, through binding to both ISE2 and ISE3, is an essential host factor for maturation of 11-kDa-encoding mRNA.

## Abstract (precis)

Human parvovirus B19 (B19V) is a human pathogen that causes severe hematological disorders in immunocompromised individuals. B19V infection has a remarkable tropism with respect to human erythroid progenitor cells (EPCs) in human bone marrow and fetal liver. During B19V infection, only one viral precursor mRNA (pre-mRNA) is transcribed by a single promoter of the viral genome and is alternatively spliced and alternatively polyadenylated, a process which plays a key role in expression of viral proteins. Our studies revealed that a cellular RNA binding protein, RBM45, binds to two intron splicing enhancers and is essential for the maturation of the small nonstructural protein 11-kDa-encoding mRNA. The 11-kDa protein plays an important role not only in B19V infection-induced apoptosis but also in viral DNA replication. Thus, the identification of the RBM45 protein and its cognate binding site in B19V pre-mRNA provides a novel target for antiviral development to combat B19V infection-caused severe hematological disorders.

## INTRODUCTION

Human parvovirus B19 (B19V), a member of the *Erythroparvovirus* genus within the *Parvoviridae* family (1), has a linear single-stranded DNA (ssDNA) genome of 5,596 nucleotides (nt) that is flanked by two identical inverted terminal repeats (ITRs) (2–4). B19V is an autonomously replicating human parvovirus with a remarkable tropism for human erythroid progenitor cells (EPCs) of the bone marrow and fetal liver (5–7). B19V infection can result in hematological disorders under a variety of circumstances. In patients with increased destruction of erythrocytes and a high demand for erythrocyte production (for example, during sickle cell anemia and hereditary spherocytosis), acute B19V infection can cause transient aplastic crisis (8–11), and in immunocompromised patients, such as AIDS patients and organ transplant recipients, B19V infection leads to persistent viremia associated with chronic anemia and pure red-cell aplasia (12–18). In fetuses of pregnant women, B19V infection can result in nonimmune hydrops fetalis and fetal death (19–23). The clinical manifestations of B19V infection in patients with transient aplastic crisis, pure red-cell aplasia, chronic anemia, and hydrops fetalis are due to the direct cytotoxicity of the virus infection (24–26), a direct outcome of the cell cycle arrest and cell death of the EPCs that host B19V replication (7, 27–30).

During B19V replication, after conversion from the ssDNA viral genome, the viral double-stranded DNA (dsDNA) replicative form (RF) genome is transcribed by the single P6 promoter. The viral precursor mRNA (pre-mRNA) is alternatively spliced and alternatively polyadenylated resulting in the generation of nine major viral mRNA transcripts that encode capsid proteins (VP1 and VP2) and nonstructural proteins (NS1, 11-kDa, and 7.5-kDa) (31–34). NS1 is the only viral protein that is essential for B19V DNA replication (35–37). However, the small nonstructural protein 11-kDa, which is localized predominantly in the cytoplasm and plays an enhancement role in viral DNA replication, is unique among parvoviruses (38). Mechanistically, the 11-kDa protein interacts with Grb2 (growth factor receptor bound protein 2) through the three SH3-binding motifs of the 11-kDa protein. This interaction disrupts the extracellular signal-regulated kinase-1 signaling and upregulates viral DNA replication (38).

Alternative processing of B19V pre-mRNA plays a key role in regulating expression of viral proteins through interactions of host RNA-binding proteins with the viral pre-mRNA (39–41). The intronic splicing enhancer 2 (ISE2), located immediately downstream of the second donor (D2), is critical for splicing of the viral pre-mRNA at D2, which controls production of the mRNAs encoding both VP2 and 11-kDa (41). We have shown that RNA binding motif protein 38 (RBM38) binds to ISE2 and executes a function in splicing of viral pre-mRNA at D2 and the second acceptor of the second intron (A2-2), which encodes mRNA encoding 11-kDa (37). However, how the RBM38 specifically regulates maturation of the 11-kDa-encoding mRNA is unknown.

RBM45, also named drb1, was recently found to be a FUS (fused-in-sarcoma) protein-interacting RNA-binding protein (42). It is localized predominately in the nucleus (43), where it plays an important role in the DNA damage response (DDR) (44). It is recruited to the DNA damage sites in a poly(ADP-ribose) (PAR)-dependent and FUS-dependent manner and promotes dsDNA break repair by preventing histone deacetylase 1 from excessive recruitment. However, under disease conditions, such as those seen with amyotrophic lateral sclerosis (ALS), frontotemporal lobar degeneration, and Alzheimer’s disease patients (45), RBM45 distributes within transactive response (TAR) DNA-binding protein 43 (TDP43)-positive cytoplasmic inclusions, where RBM45 physically associates with TDP-43 (42). Although it was initially identified as an RNA-binding protein and exhibited preferential binding to poly(C) RNA (46), the functions of RBM45 in the DDR and under these disease conditions are not mediated by any RNAs. Proteomics analysis of the RBM45 interactome has identified a number of splicing factors (47); however, the role of RBM45 in RNA-mediated functions, including mRNA splicing, has never been experimentally explored.

In this study, we took an RNA pulldown and mass spectrometry (MS) approach to identify host proteins that interact with the B19V ISE2. We found that RBM45 bound both to ISE2 and to the novel ISE3 that is located in front of the branch point of the A2-2 acceptor. We propose that RBM45 plays a role as a scaffold protein to bring the U1 snRNP that interacts with D2 and the U2 snRNP that is recruited to the A2-2 acceptor, which facilitates spliceosome formation of the second intron (D2 to A2-2 sites) of the B19V pre-mRNA.

## RESULTS

### Identification of B19V ISE2-interacting proteins.

**FIG 1:**
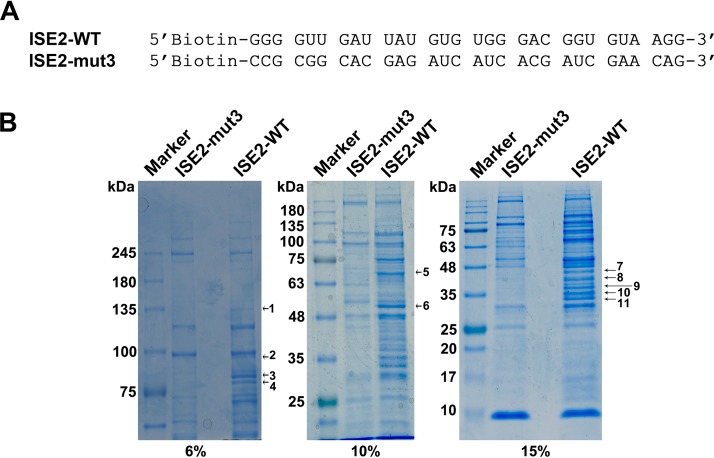
Identification of B19V ISE2-interacting proteins. (A) ISE2 wild-type (WT) and ISE2 mutant (mut3) RNA sequences. The two RNAs used to pull down ISE2-interacting proteins are listed. (B) SDS-PAGE. The proteins pulled down by the biotinylated ISE2-WT and ISE2-mut3 RNAs from nuclear lysates of UT7/Epo-S1 cells were separated by 6%, 10%, or 15% SDS-PAGE as indicated and stained with Coomassie blue to visualize proteins. The bands uniquely stained in the ISE2-WT pulldown sample but not in the ISE2-mut3 sample are indicated.

We previously reported that ISE2 is critical for the recognition of the D2 site for splicing and that the interaction of RBM38 with ISE2 promotes splicing of the intron from the D2 to the A2-2 splice sites (37, 41). Thus, we asked whether other host factors could interact with ISE2 and facilitate the alternative splicing at these sites. To this end, we synthesized wild-type (WT) ISE2 RNA (ISE2-WT) and mutant ISE2 RNA (ISE2-mut3) (37, 41) with biotin labeling at the 5′ ends (Fig. 1A) and incubated them with nuclear lysates extracted from UT7/Epo-S1 cells in the presence of poly(I·C). We then performed an RNA pulldown assay using streptavidin-conjugated agarose beads that bound biotinylated RNA molecules. Pulldown nuclear proteins were separated by sodium dodecyl sulfate-polyacrylamide gel electrophoresis (SDS-PAGE) and stained with Coomassie blue. Eleven obviously unique bands of >30 kDa in the sample pulled down by ISE2-WT, but not by ISE2-mut3 (Fig. 1B), were excised for liquid chromatography with tandem mass spectrometry (LC/MS/MS). From the MS results, we chose 22 proteins with ≥20 unique peptide hits, or the top 3 proteins (see Table S1 in the supplemental material), which are related to mRNA processing/RNA binding as analyzed by UniProt (https://www.uniprot.org/). We then performed RNA pulldown assays to confirm the binding of the 22 candidates with biotinylated RNA. We found that ISE2-WT pulled down 16 candidate proteins, whereas ISE2-mut3 did not (see Fig. S1 in the supplemental material), suggesting that these 16 proteins directly or indirectly interact with ISE2.

10.1128/mBio.00192-20.1 FIG S1 RNA pulldown and Western blotting. Biotinylated ISE2-mut3 and ISE2-WT were incubated with UT7/Epo-S1 nuclear lysates in the presence of poly(I·C) followed by pulldown by the use of streptavidin-conjugated beads. The RNA-bound proteins were analyzed by Western blotting using antibodies as indicated. A total of 4% of the nuclear lysate of UT7/Epo-S1 used for pulldown was run in the first lane as an input control. Download FIG S1, TIF file, 0.9 MB. Copyright © 2020 Wang et al. 2020 Wang et al. This content is distributed under the terms of the Creative Commons Attribution 4.0 International license.

10.1128/mBio.00192-20.3 TABLE S1 Mass spectrometry identified 32 proteins with ≥20 unique reads or with the top 3 in the band. Listed are 32 proteins identified by mass spectrometry with ≥20 unique reads or with the top 3 in the band. A total of 10 proteins (underlined) did not have a function in RNA biogenesis as analyzed by UniProt (https://www.uniprot.org/) and were not tested for RNA binding. All shRNP proteins were not tested by knockdown, as they function as negative regulators. SF3b1 is essential for cell growth, and DHX37 and FUBP1 were not successfully knocked down in UT7/Epo-S1 cells, which are marked with a star (*). ND, not determined. Download Table S1, DOCX file, 0.03 MB. Copyright © 2020 Wang et al. 2020 Wang et al. This content is distributed under the terms of the Creative Commons Attribution 4.0 International license.

### RBM45 is essential for expression of viral 11-kDa during B19V infection of CD36^+^ EPCs.

We next asked whether knockdown of the expression of these ISE2-interacting proteins affected expression of the VP2 and 11-kDa proteins. As hnRNP proteins function as negative regulators of mRNA splicing (48), we excluded all hnRNP proteins during the knockdown procedures, as well as SF3B1, which is an essential U2 snRNP component (49), and DHX37, which plays a role in ribosome biogenesis (50). We found the presence of lentivirally expressed short hairpin RNA (shRNA) specifically targeting *RBM6*, *KHSRP*, *DDX21*, *LARP7*, *PURA*, or *RBM45* (Table S2; see also Fig. S2) drastically reduced expression of 11-kDa, but not VP2, in UT7/Epo-S1 cells transfected with B19V infectious clone M20, whereas shRNA targeting *SRSF1* (*ASF/SF2*) and *DHX9* did not (Fig. S2). We met difficulties in achieving sufficient knockdown of FUBP1, which has a positive role in mRNA splicing regulation (51) (Table S1). As RBM45 was previously shown to interact with a number of splicing factors in a proteomics analysis (47) and as its functions in mRNA splicing have never been investigated, we chose RBM45 for use in exploring roles in viral mRNA splicing and 11-kDa expression.

10.1128/mBio.00192-20.2 FIG S2 Knockdown of candidate genes for expression of the 11-kDa protein and VP1/2. UT7/Epo-S1 transduced with shRNA, as indicated, and scramble shRNA (shScram)-expressing lentiviruses were transfected with M20 at 2 days posttransduction. The cells were collected at 2 days posttransfection, lysed, and run for Western blotting. Blots were probed for the VP1/2, NS1, and 11-kDa viral proteins, as well as for shRNA-targeting gene product (protein), using their respective antibodies. Blots were reprobed for β-actin as a loading control. Download FIG S2, TIF file, 1.5 MB. Copyright © 2020 Wang et al. 2020 Wang et al. This content is distributed under the terms of the Creative Commons Attribution 4.0 International license.

10.1128/mBio.00192-20.4 TABLE S2 shRNA targeting sequences used to knock down the genes listed in Fig. S2. Listed are 7 sequences of the shRNAs that were used in shRNA-expressing lentiviral vectors. Download Table S2, DOCX file, 0.01 MB. Copyright © 2020 Wang et al. 2020 Wang et al. This content is distributed under the terms of the Creative Commons Attribution 4.0 International license.

**FIG 2:**
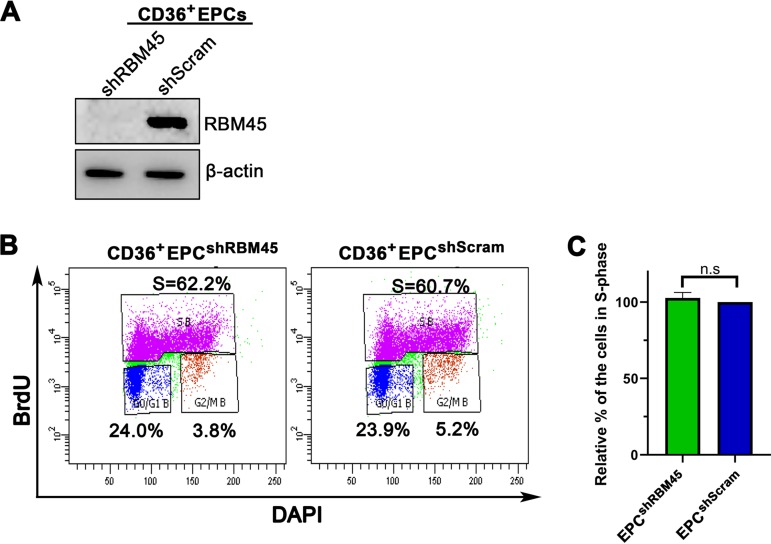
RBM45 knockdown did not alter cell cycle progression in CD36^+^ EPCs. (A) RBM45 knockdown. Lentiviruses expressing shRNAs against RBM45 were transduced into CD36^+^ EPCs. At 3 days postransduction, the cells were lysed for Western blot analysis using an anti-RBM45 antibody. The blots were reprobed for β-actin. (B and C) Cell cycle analysis. CD36^+^ EPCs were transduced with shScramble (shScram) or shRBM45. A BrdU incorporation assay was used to track *de novo* DNA synthesis. The cells were processed and analyzed for cell cycle progression using flow cytometry. (B) Representative histograms showing cell cycle analysis of the control CD36^+^ EPC^shScram^ and CD36^+^ EPC^shRBM45^ cells. DAPI, 4′,6-diamidino-2-phenylindole. (C) Values representing relative fold changes of the cell population in S phase were calculated for CD36^+^ EPCs. Each experiment was repeated three times for the calculation of means and standard deviations. n.s, no statistical significance (*P* > 0.05).

**FIG 3:**
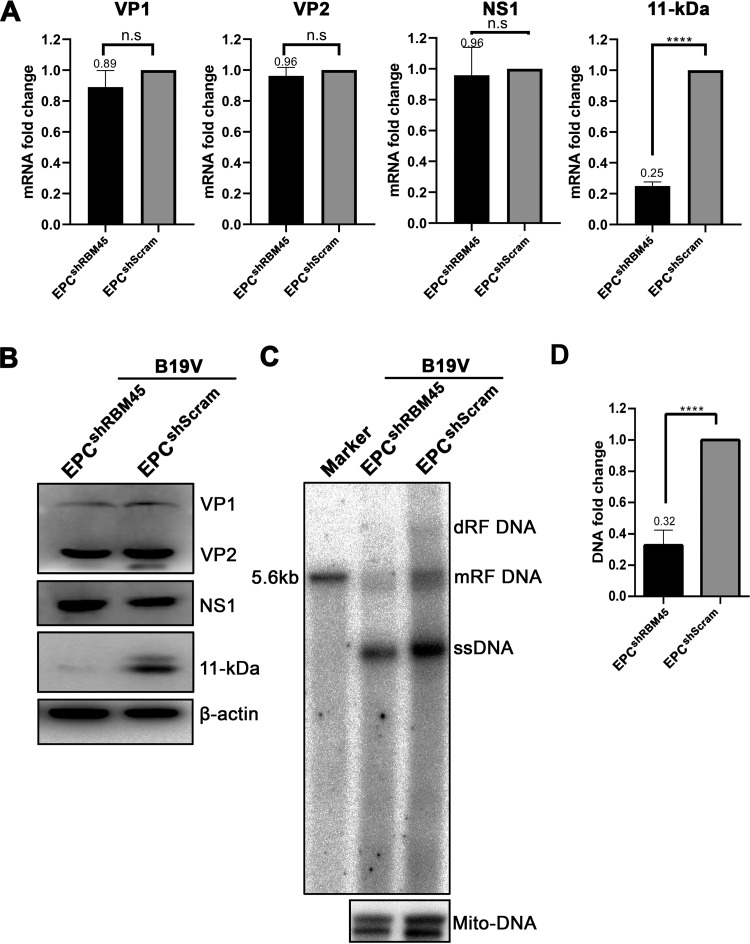
RBM45 regulated 11-kDa protein expression in CD36^+^ EPCs. CD36^+^ EPCs transduced with shRBM45-expressing and scramble shRNA (shScram)-expressing lentiviruses were infected with B19V at 2 days postransduction. The cells were collected at 2 days postinfection for analyses. (A) RT-qPCR. Total RNA was extracted. cDNAs reverse transcribed from the RNAs were used for qPCR of each viral mRNA, as indicated, using specific primers and probes for each viral mRNA as described previously (37). The quantified viral mRNA level was normalized to the level of β-actin mRNA. The values representing viral mRNA from B19V-infected CD36^+^ EPCs transduced with shScram were used as controls and arbitrarily set to 1. (B) Western blotting. CD36^+^ EPCs were collected at 2 days postinfection, lysed, and run for Western blotting. Blots were probed for the VP1, VP2, NS1, and 11-kDa proteins, using their respective antibodies. Blots were reprobed for β-actin as a loading control. (C and D) Southern blotting. At 2 days postinfection, Hirt DNA was extracted from CD36^+^ EPCs for Southern blot analysis. (C) The blots were probed with the M20 probe (top) and the mitochondrial DNA probe (Mito-DNA) (bottom), respectively. Representative blots are shown. dRF, mRF, and ssDNA, double replicative form, monomer replicative form, and single-stranded DNA, respectively. A 10-ng volume of SalI-digested M20 was used as a size marker of 5.6 kb. (D) The intensity of the RF DNA band was quantified and normalized to the level of the mitochondrial DNA (Mito-DNA) of each sample. The value representing viral RF DNA in B19V-infected CD36^+^ EPCs transduced with shScram was arbitrarily set to 1. Values representing relative fold change of the viral RF DNA in B19V-infected CD36^+^ EPCs transduced with shRBM45 are shown with averages and standard deviations from three repeated experiments.

RBM45 knockdown did not obviously alter the cell cycle progression of CD36^+^ EPCs (Fig. 2B and C). We tested the role of RBM45 in B19V viral mRNA processing in B19V-infected CD36^+^ EPCs. Control shScramble (shScram)-transduced CD36^+^ EPCs and RBM45 knockdown CD36^+^ EPC^shRBM45^ were infected with B19V, and the cells were collected for total RNA extraction at 48 h postinfection, followed by analysis of viral mRNA (VP1, VP2, NS1, and 11-kDa) using reverse transcription-quantitative PCR (RT-qPCR). The results showed that RBM45 knockdown significantly decreased levels of 11-kDa coding mRNA by 4-fold, but the level of other viral mRNAs remained unaffected (Fig. 3A). We also tested the viral proteins in CD36^+^ EPC^shRB45^ using Western blotting. The results showed that the level of the 11-kDa protein, but not VP1, VP2, and NS1, was significantly decreased in B19V-infected CD36^+^ EPCs upon RBM45 knockdown (Fig. 3B). As 11-kDa plays an enhancement role in B19V DNA replication (37), we asked whether RBM45 knockdown decreased viral DNA replication via decreasing 11-kDa protein expression. We infected CD36^+^ EPC^shRBM45^ with B19V. At 48 h postinfection, the cells were collected and examined for DNA replication by Southern blotting. The results showed that RBM45 knockdown significantly decreased viral DNA replication by >3-fold compared with that seen in the cells treated with scramble shRNA (Fig. 3C and D).

Taking all these results together, we demonstrated that RBM45 regulates 11-kDa protein expression through decreasing splicing of the viral pre-mRNA for production of the 11-kDa-encoding mRNA in B19V-infected CD36^+^ EPCs. We also confirmed that the RBM45 plays an important role in B19V DNA replication through the regulation of 11-kDa protein expression (37).

### Identification of a novel intronic splicing enhancer in B19V pre-mRNA that is bound by RBM45.

**FIG 4:**
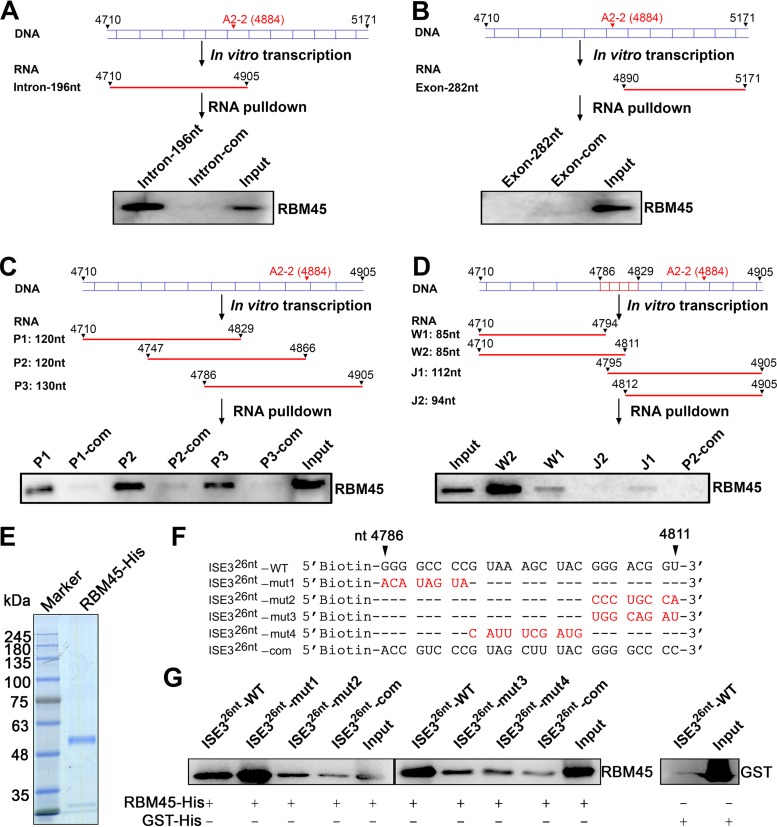
Identification of a novel intronic splicing enhancer in B19V pre-mRNA that was bound by RBM45 specifically. (A to D) Schematic representations of *in vitro* transcription and RNA pulldown assay. The B19V DNA region surrounding the A2-2 site is shown. Biotin-labeled *in vitro*-transcribed RNA (shown in red) was captured by streptavidin-conjugated agarose beads, followed by incubation with UT7/Epo-S1 nuclear lysates. RNA-bound proteins were eluted and analyzed by Western blotting. (A) B19V DNA (nt 4710 to 4905) was used as a template to transcribe RNA (named 11-kDa intron), while its cRNA (named intron com) was transcribed as a negative control. (B) B19V DNA (nt 4890 to 5171) was used as a template to transcribe RNA (named 11-kDa exon), while its cRNA (named exon com) was transcribed as a negative control. (C) B19V DNA (nt 4710 to 4905) was *in vitro* transcribed to generate 3 overlapped small RNA segments termed RNA P1 (nt 4710 to 4829), P2 (nt 4747 to 4866), and P3 (nt 4786 to 4905). cRNA P1, P2, and P3 (complementary P1 [P1-com], P2-com, and P3-com, respectively) were transcribed as negative controls. (D) B19V DNA (nt 4710 to 4905) was further *in vitro* transcribed to generate 4 small segments termed RNA W1 (nt 4710 to 4794), W2 (nt 4710 to 4811), J1 (nt 4795 to 4905), and J2 (nt 4812 to 4905), while P2 cRNA (P2-com) was transcribed as a negative control. (E to G) RNA pulldown assay using biotinylated ISE3^26nt^ WT and its mutants. Recombinant RBM45-His protein (E) was incubated with the corresponding biotinylated RNA oligonucleotides (F), pulled down with streptavidin-conjugated beads, and analyzed by Western blotting for RBM45 (G). For panel E, A 6-μg volume of the RBM45-His protein was run on an SDS-10% PAGE gel and then stained with Coomassie brilliant blue dye. Complementary ISE3^26nt^ (ISE3^26nt^-com) and GST-His served as the negative RNA and protein controls, respectively.

In order to determine the mechanism underlying the RBM45 regulation of 11-kDa-encoding mRNA production, we looked for the RNA sequence that was bound by RBM45. As RBM45 specifically targeted 11-kDa expression, which is encoded from viral mRNA spliced at the A2-2 acceptor, we looked for the RBM45-binding site in a viral RNA sequence near the A2-2 acceptor using an *in vitro* RNA pulldown assay. We synthesized a large portion of the intron (nt 4710 to 4905) before the A2-2 acceptor *in vitro*, labeled it with biotin, and used it to pull down RBM45 in nuclear lysates extracted from UT7/Epo-S1 cells. Similarly, we biotinylated the B19V 11-kD exon mRNA (nt 4890 to 5171) to perform the RNA pulldown assay with the cell lysates. The results showed that RBM45 bound to the intron part of the B19V RNA from nt 4710 to nt 4905 (Fig. 4A) but not the exon RNA (Fig. 4B). To further identify the RBM45-binding site, we performed *in vitro* transcription of overlapped RNAs in the region of nt 4710 to 4905 and used them in the RNA binding assay. We identified the left end of the RBM45-binding sequence at nt 4786 (Fig. 4C). We then used a similar strategy to determine the right end of the RBM45 binding site at nt 4811 (Fig. 4D). Finally, we narrowed down the binding sequence to nt 4786 to 4811 (26 nt), which we named intronic splicing enhancer 3–26 nt (ISE3^26nt^).

We next asked whether purified RBM45 protein could bind to ISE3^26nt^ of 26 nt. We purified His-tagged RBM45 (Fig. 4E) and synthesized 5′-biotin WT ISE3^26nt^ and four mutant ISE3^26nt^ (ISE3^26nt^-mut1, mut2, mut3, and mut4) RNAs (Fig. 4F). The ISE3^26nt^ complementary (ISE3^26nt^-com) RNA and His-tagged glutathione *S*-transferase (GST-His) protein served as the negative RNA and protein controls, respectively. Upon incubation of the six RNA molecules with purified RBM45 or GST in the presence of poly(I·C), we pulled down the RNAs using streptavidin-coated beads and found that ISE3^26nt^-WT pulled down much more RBM45 than ISE3^26nt^-mut2, ISE3^26nt^-mut3, and ISE3^26nt^-mut4 (Fig. 4G). Of note, ISE3^26nt^-mut1 bound to more RBM45 than ISE3^26nt^-WT, indicating that the 8-GC sequence (GGGGCCCC) at the 5′ end of ISE3^26nt^ was not necessary for the binding of ISE3^26nt^ with RBM45.

Collectively, the data confirmed the essentiality of ISE3, with 5′-GUA AAG CUA CGG GAC GGU-3′, the 18 nucleotides of ISE3^26nt^, representing a novel ISE of B19V pre-mRNA that binds RBM45.

### RBM45 specifically binds to ISE3 at high affinity under *in vitro* conditions.

**FIG 5:**
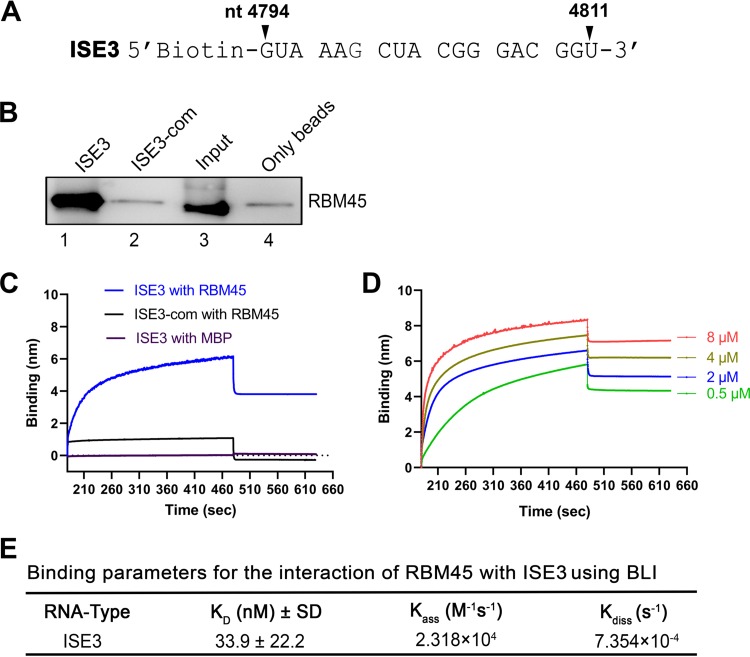
RBM45 specifically bound to ISE3 under *in vitro* conditions. (A) ISE3 RNA sequence. The ISE3 RNA shown was used in the binding assay. (B) RNA pulldown assay. Biotinylated ISE3 and complementary ISE3 (ISE3-com) were incubated with recombinant RBM45-His protein and were then pulled down by using streptavidin-conjugated beads. Eluted RNA-bound proteins were analyzed by Western blotting for RBM45. The recombinant RBM45-His protein (Input) and the no RNA-pulldown sample were loaded in lanes 3 and 4 as input and bead only controls, respectively. (C to E) Biolayer interferometry. (C) Comparison of binding affinities of the RBM45 protein (2 μM) for the ISE3 and ISE3-com RNAs, respectively. Maltose-binding protein (MBP-His) was used as a negative protein control. (D) BLI sensorgrams showing association and dissociation of the RBM45 protein with ISE3 at different concentrations over time as indicated. (E) Binding parameters used to calculate *K_D_* values (ratios of dissociation and association rate constants). The binding experiments were repeated at least three times for calculating the means and standard deviations (SD), by using various concentrations of the protein.

In order to confirm whether the presence of ISE3 was sufficient for binding to RBM45 and to determine the specificity of the interaction, we used biotinylated ISE3 (Fig. 5A) and purified RBM45 in the RNA pulldown assay. The results showed that ISE3 pulled down RBM45 at a much higher level of efficiency than its complementary sequence, ISE3-com (Fig. 5B). Furthermore, to determine the affinity of binding of RBM45 with ISE3, we used biotinylated ISE3 or ISE3-com in an *in vitro* binding assay based on biolayer interferometry (BLI). The BLI assays showed that RBM45 bound to ISE3 but not the His-tagged maltose-binding protein (MBP-His) control and that RBM45 also did not bind to ISE3-com control RNA (Fig. 5C). Then, we used various concentrations of RBM45 to determine the binding affinity for RBM45 with ISE3. The results showed that there were related increases in the levels of binding of ISE3 and RBM45 as the concentration of RBM45 increased (Fig. 5D). The equilibrium dissociation constant (*K_D_*) corresponding to the binding between ISE3 and RBM45 was calculated to be 33.9 ± 22.2 nM (Fig. 5E). Taking the results together, we had demonstrated that RBM45 specifically binds to ISE3 of B19V pre-mRNA at high affinity.

### The RRM2-HOA domain of RBM45 is largely responsible for binding to ISE3 of B19V pre-mRNA, overexpression of which decreases expression of 11-kDa.

**FIG 6:**
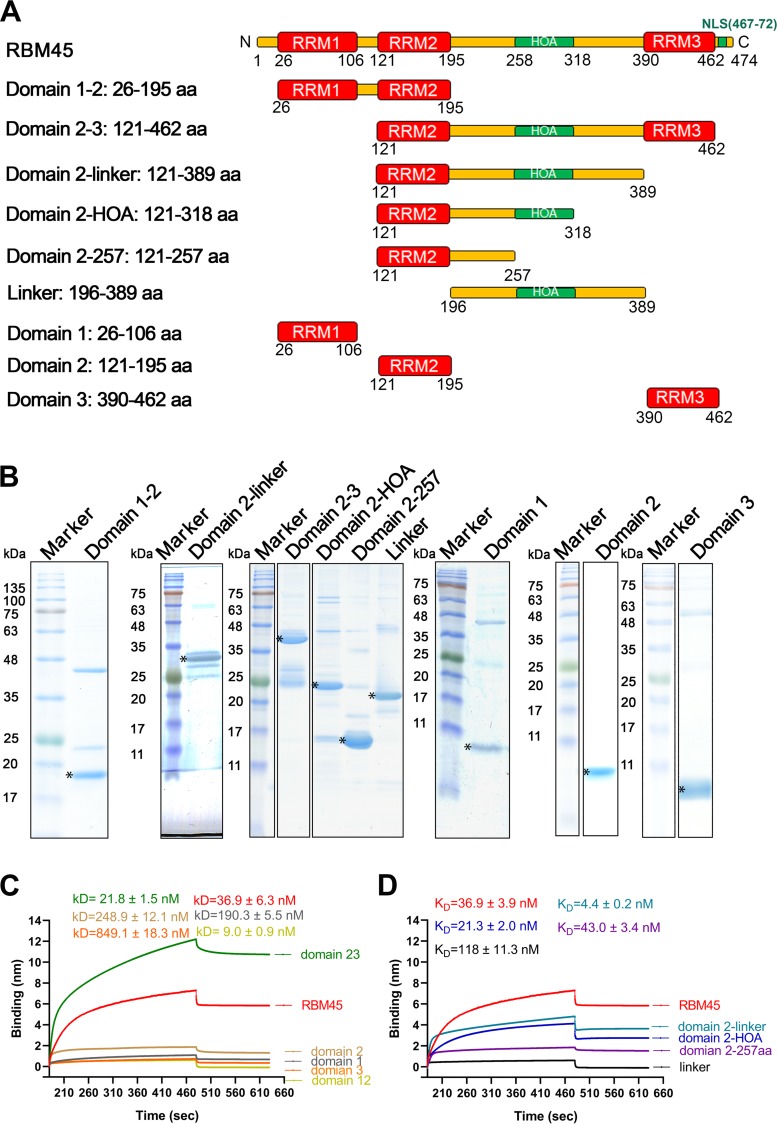
The RRM2-HOA domain of RBM45 was largely responsible for binding to ISE3 of B19V pre-mRNA. (A) Domain structures of RBM45. The RNA recognition motif (RRM) domains are shown in red. The homo-oligomer assembly (HOA) domain and nuclear localization sequence (NLS; residues 469 to 472) are shown in green. Linker regions between RRMs are diagrammed in yellow. (B) Purification of truncated RBM45 proteins. Purified recombinant proteins (6 μg) were separated on an SDS-10% or 15% PAGE gel and stained with Coomassie brilliant blue dye. Asterisks denote the truncated proteins. (C and D) Biolayer interferometry (BLI) analysis. We used BLI to measure the interactions of ISE3 with different truncated and full-length forms of RBM45. (C) Domain 1–2 (aa 26 to 195), domain 2–3 (aa 121 to 462), domain 1 (aa 26 to 106), domain 2 (aa 121 to 195), and domain 3 (aa 390 to 462) of RBM45 were analyzed for their binding capability with ISE3. Domain 2–3 (aa 121 to 462) was found to be the key domain for RBM45 binding to ISE3. (D) Data representing domain 2-linker (aa 121 to 389) and 2-HOA (aa 121 to 318) were analyzed and compared with data representing binding to the full-length RBM45. *K_D_* values are shown for each binding curve. The binding experiments were repeated at least three times for calculating means and standard deviations (SD).

We then asked which domain of RBM45 was responsible for the binding to ISE3. As shown in Fig. 6A, RBM45 contains three RNA recognition motif (RRM) domains, namely, RRM1, RRM2, and RRM3 (shown in red in Fig. 6A), a homo-oligomer assembly (HOA) domain (shown in green), and the linker between them (shown in yellow) (42). We purified 9 different truncated RBM45 proteins tagged with His (Fig. 6B) to test their binding with ISE3 using the BLI assay. First, we tested the binding of domains 1, 2, 3, 1–2, and 2–3 with ISE3. The results showed that the level of affinity of domain 2–3 for ISE3 (21.8 ± 1.5 nM) was similar to that seen with full-length RBM45 (36.9 ± 6.3 nM) (Fig. 6C). Domain 2-HOA and the domain corresponding to amino acids (aa) 2 to 257 had *K_D_* values for ISE3 of 21.3 ± 2.0 nM and 43.0 ± 3.4 nM, respectively (Fig. 6D). Thus, we determined that the RRM2-HOA domain (aa 121 to 318) is the key domain for binding of RBM45 with ISE3.

**FIG 7:**
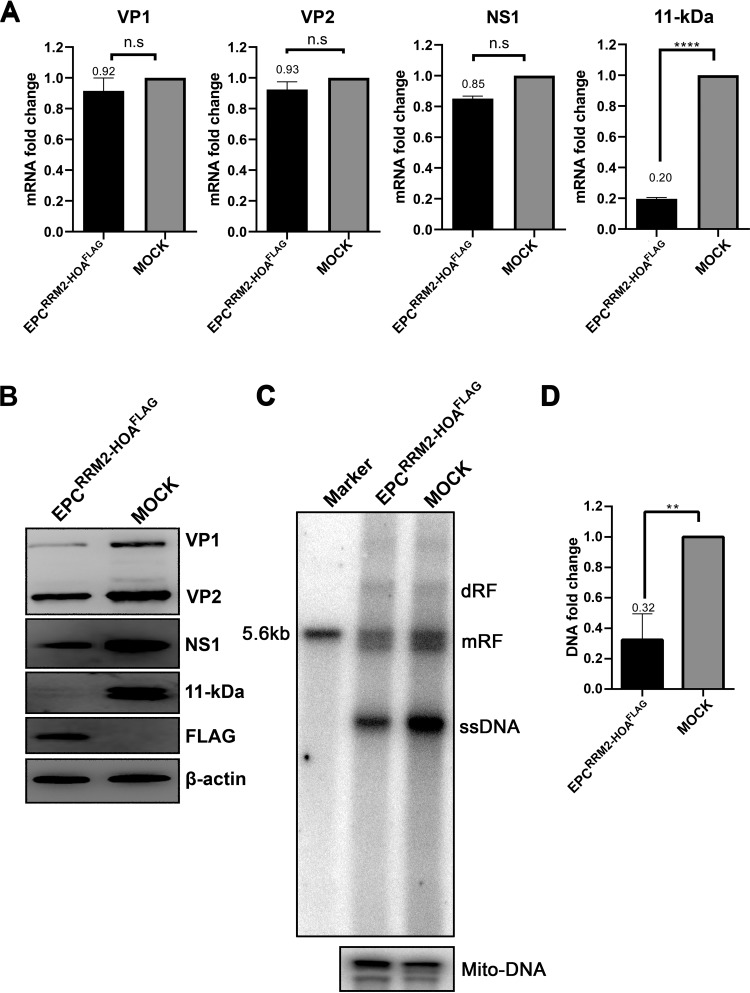
Expression of the ISE3-binding domain of RBM45 decreased expression of 11-kDa at the levels of both mRNA and protein. CD36^+^ EPCs were transduced with a lentivirus expressing RBM45-RRM2-HOA. At 3 days postransduction, the cells were infected with B19V. After 48 h, the cells were collected for analyses of expression of viral mRNAs and proteins and of viral DNA replication. (A) RT-qPCR. Total RNA was extracted from CD36^+^ EPCs and reverse transcribed to generate cDNA, which was used for quantification of the viral mRNAs that encode the VP1, VP2, NS1, and 11-kDa proteins by the use of qPCR. The quantified viral mRNA levels were normalized to the level of β-actin mRNA, and the values representing mRNAs quantified from B19V-infected control CD36^+^ EPCs were arbitrarily set at 1. (B) Western blotting. The cells were lysed and analyzed by Western blotting. The blots were probed for the VP1/2, NS1, and 11-kDa proteins, using their respective antibodies. β-Actin was reprobed as a loading control. (C and D) Southern blotting. At 2 days posttransfection, Hirt DNA samples were prepared from CD36^+^ EPCs and were subjected to Southern blot analysis. SalI-digested pM20 (10 ng) was loaded as a size marker. (C) The blots were probed with the M20 probe (top) and the Mito-DNA probe (bottom). Representative blots are shown. dRF, mRF, and ssDNA, double replicative form, monomer replicative form, and single-stranded DNA, respectively. (D) The intensity of the RF DNA band was quantified and normalized to the level of the mitochondrial DNA (Mito-DNA) of each sample. The value representing viral RF DNA in B19V-infected CD36^+^ EPCs transduced with shScram was arbitrarily set to 1. Relative fold change of the viral RF DNA in B19V-infected CD36^+^ EPCs transduced with shRBM45 is shown with averages and standard deviations of results from three repeated experiments.

Having identified the ISE3-binding domain of RBM45 (RRM2-HOA), we attempted to overexpress it in CD36^+^ EPCs to look for an effect of expression of 11-kDa on B19V infection. We observed that overexpression of RRM2-HOA significantly decreased the levels of 11-kDa-encoding mRNAs (Fig. 7A) and the 11-kDa protein (Fig. 7B), as well as viral DNA replication (Fig. 7C and D). This result strongly suggested that the RRM2-HOA domain competes with endogenous RBM45 and diminishes the function of RBM45 in expression of 11-kDa-encoding mRNA.

### A B19V duplex genome clone (M20) that harbors silent mutations of ISE3 expresses a low level of the 11-kDa-encoding mRNA that is spliced at A2-2, resulting in a low level of expression of 11-kDa in transfected cells.

Having determined that RBM45 specifically binds to ISE3, we asked whether silent mutations of ISE3 in B19V infectious clone M20 would have an effect on expression of 11-kDa. Transfection of M20 into UT7/Epo-S1 cells confers viral DNA replication and production of virions that are infectious in EPCs (52).

**FIG 8:**
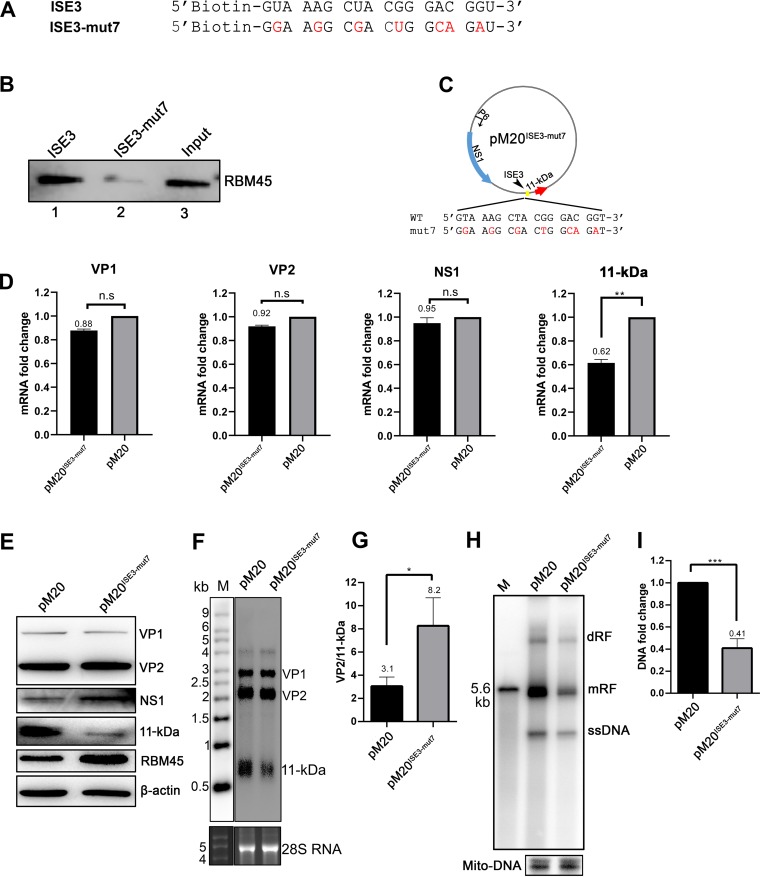
A B19V M20 infectious clone that harbors silent mutations of ISE3 expressed a low level of the mRNA spliced at the D2 and A2-2 sites and of 11-kDa protein in transfected cells. (A) ISE3 and ISE3 mutant 7 (ISE3-mut7) RNA sequences. The two RNAs shown were used in the binding experiment. (B) RNA pulldown assay. Biotinylated ISE3 and ISE3-mut7 were incubated with RBM45-His protein and then pulled down by using streptavidin beads. The RNA-bound proteins were eluted and analyzed by Western blotting for RBM45, as indicated. Approximately 4% of the RBM45-His protein was loaded as input (lane 3). (C) Diagram of pM20^ISE3-mut7^. Silent mutations in the ISE3 of the M20 clone (pM20^ISE3-mut7^) are diagrammed and shown with 7 mutations in red, while the coded amino acids of the VP1/2 protein remained unchanged. (D and E) RNA and protein analyses. UT7/Epo-S1 cells were transfected with either M20^ISE3-mut7^ or the parent M20 clone. (D) RT-qPCR. At 2 days posttransfection, total RNA was extracted from treated UT7/Epo-S1 cells and reverse transcribed. cDNA was used for quantification of the mRNAs that encode the VP1, VP2, NS1, and 11-kDa proteins by the use of qPCR. Quantified viral mRNA levels were normalized to the level of β-actin mRNA, and the value representing mRNA quantified from M20-transfected control UT7/Epo-S1 cells was arbitrarily set to 1. (E) Western blotting. At 2 days posttransfection, cells were lysed and run for Western blotting for expression of viral proteins as indicated. β-Actin was used as a loading control. (F and G) Northern blotting of viral mRNAs. At 2 days posttransfection, total RNA was extracted from treated UT7/Epo-S1 cells and was subjected to Northern blotting using a *cap* probe. The detected viral mRNA bands are indicated. The Millennium RNA marker (M; Invitrogen) was loaded and blotted with a probe that hybridizes to it. Ethidium bromide staining of the RNA gel shows the 28S ribosome RNA. (F) The levels of VP2 and 11-kDa mRNAs were quantified, and the ratio of VP2/11-kDa mRNA was calculated from three independent experiments. (H and I) Southern blotting (viral DNA replication). At 2 days posttransfection, Hirt DNA samples were prepared from UT7/Epo-S1 cells and were subjected to Southern blot analysis. The results of pM20 digestion performed with SalI were loaded as a size marker. (H) The blots shown were probed with the M20 probe (top) and the Mito-DNA probe (bottom) and are representative. dRF, mRF, and ssDNA, double replicative form, monomer replicative form, and single-stranded DNA, respectively. (I) The intensity of RF DNA band was quantified and normalized to the level of the mitochondrial DNA (Mito-DNA) of each sample. The value representing viral RF DNA in UT7/Epo-S1 cells transfected with pM20 was arbitrarily set to 1. Relative fold change of the viral RF DNA in UT7/Epo-S1 cells transfected with pM20^ISE3-mut7^ is shown with averages and standard deviations of results from three repeated experiments.

We made ISE3 mutants that have silent mutations without a coding change, tested their binding with RBM45 *in vitro*, and determined that mutant ISE3-mut7 (Fig. 8A) pulled down RBM45 poorly (Fig. 8B). We introduced ISE3-mut7 into infectious clone pM20, which resulted in pM20^ISE3-mut7^ remaining a WT VP2 open reading frame (ORF) (Fig. 8C). pM20 and pM20^ISE3-mut7^ were transfected into UT7/Epo-S1 cells for analysis of the viral mRNAs and viral proteins, as well as viral DNA replication. The results showed that 11-kDa mRNA was expressed at significantly lower levels in pM20^ISE3-mut7^-transfected UT7/Epo-S1 cells than in pM20-transfected cells (Fig. 8D), which resulted in less 11-kDa protein expression (Fig. 8E). The poor splicing of the 11-kDa-encoding mRNA from the A2-2 site was further confirmed by a direct analysis of the VP2-encoding and 11-kDa-encoding mRNA by Northern blotting (Fig. 8F). And the result showed that splicing at the A2-2 site (as shown by the VP2/11-kDa mRNA ratio) was decreased by 2.6-fold (Fig. 8G). In contrast, both mRNA expression and protein expression of VP1/2 and NS1 were not affected (Fig. 8D and E). In addition, Southern blot analysis of Hirt DNA extracted from transfected cells showed that silent mutations of ISE3 in infectious clone M20 significantly decreased B19V DNA replication (by ∼2.5-fold for the monomer replicative form [mRF] DNA) compared with the results seen with the counterpart WT M20 clone (Fig. 8H and I), which was again in line with the previous observation that 11-kDa plays a role in viral DNA replication (38).

Overall, our results demonstrated that the B19V duplex genome clone that harbored silent mutations of ISE3 expressed a low level of the mRNA spliced from the D2 to the A2-2 sites, which resulted in less 11-kDa expression and viral DNA replication in transfected cells.

### RBM45 localizes within the viral DNA replication centers.

**FIG 9:**
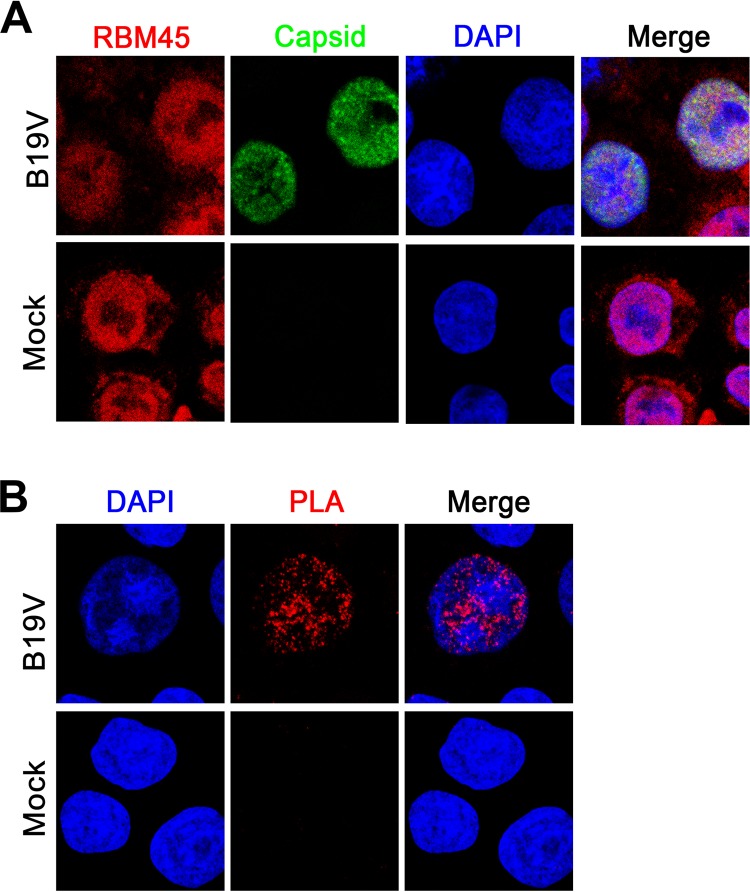
RBM45 was colocalized within the viral DNA replication centers indicated by capsid detection. CD36^+^ EPCs were mock infected or infected with B19V. At 24 h postinfection, the cells were analyzed by immunofluorescence assay. (A) Confocal colocalization. Mock-infected or B19V-infected CD36^+^ EPCs were costained and incubated with rabbit anti-RBM45 and mouse anti-B19V capsid antibodies followed by Alexa Fluor 594-conjugated and FITC-conjugated secondary antibody. (B) Proximity ligation assay (PLA). Infected cells were costained with rabbit anti-RBM45 and mouse anti-B19V capsid antibodies, followed by a proximity ligation assay, which detected two proteins detected by two labeled secondary antibodies in close proximity (∼40 nm apart). Images were taken with a Leica TCS SPE confocal microscope at ×63 magnification. Nuclei were stained with DAPI.

Next, we looked at the colocalization of the RBM45 with the viral DNA replication centers. As the viral capsid is colocalized with the replicating viral genome in the B19V replication center as previously published (53), we used an anti-capsid antibody to localize the virus replication centers. We observed precise form of colocalization of the anti-RBM45 staining (green) with the anti-capsid antibody staining in B19V-infected CD36^+^ EPCs at 48 h postinfection (Fig. 9A). This colocalization was further confirmed by a proximity ligation assay (PLA) using moue anti-capsid and rabbit anti-RBM45 antibodies (Fig. 9B), indicating that RBM45 localizes within the virus replication centers in B19V-infected EPCs.

### RBM45 specifically binds to ISE2 at high affinity *in vitro*.

**FIG 10:**
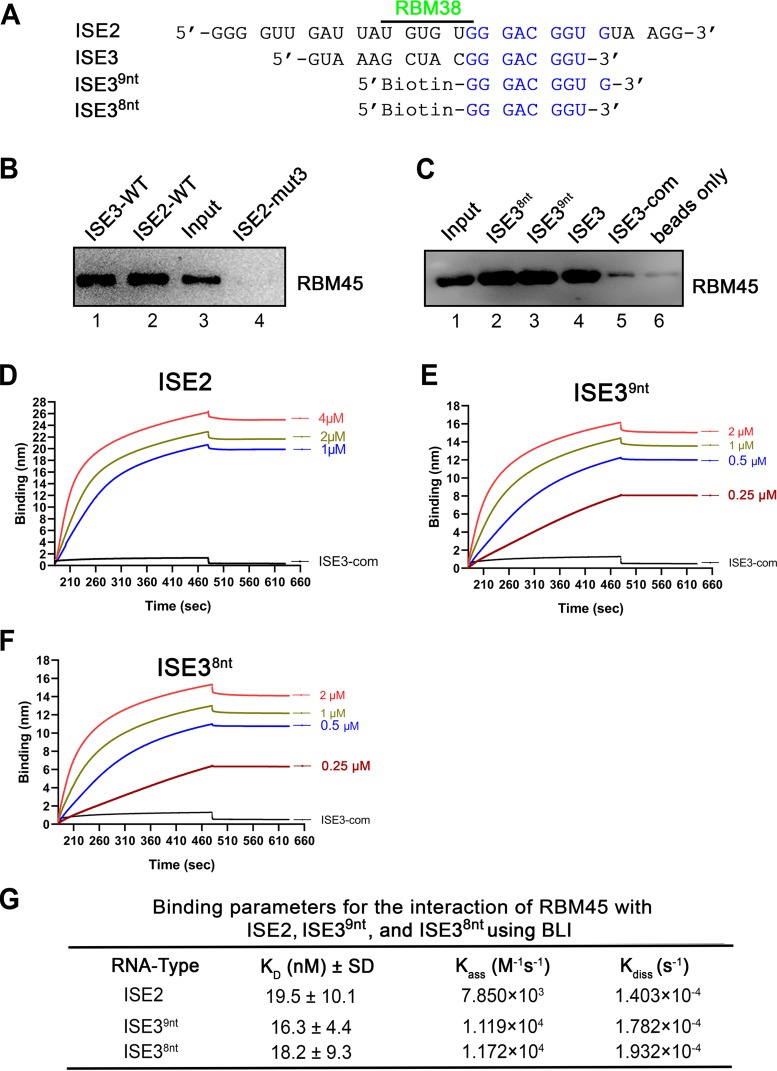
ISE2 specifically binds the RBM45 protein under *in vitro* conditions. (A) Alignment of the ISE2 and ISE3 sequences. ISE2 and ISE3 share the 8 nucleotides 5′-GGG ACG GU-3′ indicated in blue. (B and C) RNA pulldown assay. Biotinylated ISE2 and ISE3 and ISE2-mut3 (negative control) (B) and biotinylated ISE3^8nt^, ISE3^9nt^, ISE3, and ISE3-com (C) were incubated with recombinant RBM45-His protein and then pulled down by using streptavidin-conjugated beads. Eluted RNA-bound proteins were analyzed by Western blotting for RBM45. A 3-μg volume of the recombinant RBM45-His protein was loaded as the input. (D to F) Biolayer interferometry (BLI). Data represent results of comparisons of binding affinities of the RBM45 protein at the indicated concentration for ISE2 RNA (D), ISE3^9nt^ (E), and ISE3^8nt^ (F). BLI sensorgrams show association and dissociation of the RBM45 protein with ISE2 RNA (D), ISE3^9nt^ (E), and ISE3^8nt^ (F) at different concentrations over time as indicated. (G) *K_D_* determination. Binding parameters used to calculate *K_D_* values and ratios of dissociation (*K*_ass_) and association (*K*_diss_) rate constants. The binding experiments were repeated at least three times for calculating the means and standard deviations (SD), by using various concentrations of the protein.

RBM45 was initially identified in the pulldown assay using biotinylated ISE2 (Fig. 1); therefore, we tested whether RBM45 directly interacts with ISE2 (Fig. 10A). Biotinylated ISE2 and ISE3 were both able to pull down purified RBM45 but not the ISE2-mut3 control RNA (Fig. 10B). We also determined the binding affinity of RBM45 with ISE2 using BLI assays. The assays showed that RBM45 bound to ISE2, but not the ISE3-com control (Fig. 10D). The *K_D_* values representing the binding affinity of RBM45 with the ISE2 were calculated to be 19.5 ± 10.1 nM (Fig. 10G).

Notably, ISE2 contains the last 8 nucleotides (5′-GGGACGGU-3′) of ISE3 (ISE3^8nt^) (Fig. 10A), and both biotinylated ISE3^8nt^ and the maximally shared 9 nucleotides (5′-GGGACGGUG-3′ [ISE3^9nt^]) of RNAs pulled down RBM45 but not the ISE3-com control RNA (Fig. 10C). The binding affinity of RBM45 with the core ISE3^8nt^ and the extended ISE3^9nt^ was determined at various concentrations of RBM45 using BLI assays (Fig. 10E and F). The BLI results showed that RBM45 bound to both ISE3^8nt^ and ISE3^9nt^ at high affinity with *K_D_* values of 18.2 ± 9.3 nM and 16.3 ± 4.4 nM, respectively (Fig. 10G), suggesting that this octanucleotide sequence (ISE3^8nt^) represents the core RNA binding sequence of RBM45.

## DISCUSSION

**FIG 11:**
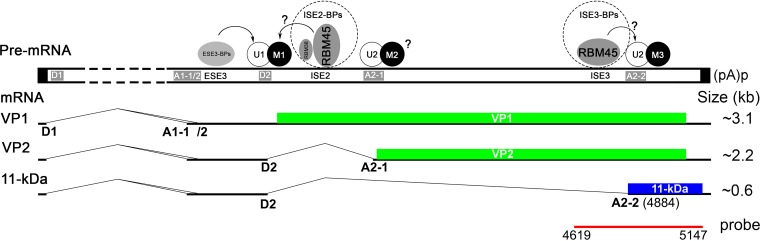
A proposed model of the role of RBM45 in B19V pre-mRNA processing. RBM45 is a scaffold protein that functions to bring the 5′ and 3′ splice sites into close proximity. The B19V pre-mRNA is depicted, with *cis* elements of D2 donor, A2-1/2 acceptors, and exon splicing enhancers (ESE) and intron splicing enhancers (ISE) indicated, together with interacted splicing factors, U1, U2, RBM45, and potential mediator proteins (M). Spliced mRNAs that encode VP1, VP2, and 11-kDa protein are diagrammed, with the dashed lines indicating introns. U1 and U2 represent U1-small nuclear ribonucleoprotein (snRNP) and U2-snRNP complexes, and M represents protein mediators associated with U1-snRNP and U2-snRNP complexes. Dotted circles represent ISE-binding proteins (ISE2/3-BPs). Question marks indicate unknown factors. The approximate size of each mRNA is indicated, and the *cap* probe for Northern blotting is diagrammed.

In this study, we revealed a novel function of RBM45 in the maturation of the 11-kDa mRNA of B19V. RBM45 specifically binds to ISE3 (5′-GUA AAG CUA CGG GAC GGU-3′), which is 72 nt upstream of the A2-2 splice acceptor. RBM45 also binds to ISE2, located immediately downstream of D2. Importantly, knockdown of RBM45 exhibits a phenotype in splicing of the 11-kDa-encoding mRNA only and not that of the VP2-encoding mRNA, highlighting the specific role of RBM45, which is similar to the role of the RBM38 (37), in splicing of viral pre-mRNA at the A2-2 acceptor for production of the 11-kDa-encoding mRNA. We propose an RBM45 working model (Fig. 11) in which RBM45 functions as a scaffold protein that facilitates the interaction between the U1 snRNP that binds to the D2 and the U2 snRNP that binds to the A2-2 acceptor, which defines the intron between the D2 and A2-2 splice sites.

In the ISE2 pulldown assay, we confirmed the presence of 16 proteins interacting with ISE2 *in vitro* (see Fig. S1 in the supplemental material), among which DHX9, KHSRP, and 4 hnRNP proteins (hnRNP H1, hnRNP D, hnRNP A3, and hnRNP H3) were found to interact with RBM45 in a previous study using immunoprecipitation-coupled mass spectrometry (47). Further knockdown of KHSRP, RBM6, DDX21, LARP7, or PURA revealed a clear decrease in 11-kDa expression (Fig. S2). As hnRNPs play notable roles in negative regulation of mRNA splicing (48), we speculate that RBM45 may function as a hub to regulate splicing of 11-kDa-encoding mRNA through interacting with other ISE2-binding proteins (ISE2-BPs), e.g., KHSRP, and/or U1-specific U1 proteins (U1A, U1C, or U70K) at the 5′ splice site and with RBM45-interacting proteins and/or U2 proteins (e.g., SF3B1) at the 3′ splice site, facilitating the interaction of U1 snRNP and U2 snRNP with the 5′ and 3′ splice sites, respectively, and defining the intron from D2 to A2-2 (Fig. 11). SF3B1 was identified in the ISE2 interactome (see Table S1 in the supplemental material), and the identification was confirmed by the RNA pulldown assay (Fig. S1). SF3B1 is a part of the SF3B complex, which is essential for assembly of the U2 snRNP and for pre-mRNA splicing (54, 55). The SF3B complex is required for assembly of the spliceosome A complex formed by stable binding of U2 snRNP to the branch point site within pre-mRNA, which is sequence independent (56). mRNA splicing involves about 150 proteins in the spliceosome, including RNA binding motif proteins (57), e.g., RBM17, a component of the core spliceosomal complex U2 snRNP (58, 59) that is abundant in intermediate complex A (58), and a number of hnRNP proteins (47). Our study determined that ISE3 is proximal to 3′ splice site A2-2 and is specifically bound by RBM45. More importantly, RBM45 plays a role in splicing of viral pre-mRNA only at the A2-2 site (for production of 11-kDa-encoding mRNA); thus, further studies should be focused on the interactions of RBM45 with protein components in the U2 snRNP.

On the other hand, we previously identified that RBM38, an erythroid-specific RNA-binding protein previously implicated in splicing (60, 61), specifically interacts with ISE2 through the “UGUGUG” motif and also plays an essential role in production of the 11-kDa-encoding mRNA (37), as RBM45 does. The *K_D_* values of RBM45 with ISE2 and ISE3 are 19.5 and 33.9 nM, respectively, suggesting RBM45 has a similar affinity with both ISE2 and ISE3. This indicates RBM38 and RBM45 likely synergistically play a role in recognition of the intron between D2 and A2-2, but not of the intron between D2 and A2-1 by facilitating binding of U1 snRNP to D2. However, a direct interaction between RBM38 and RBM45 was not confirmed (data not shown). The key RBM45 binding motif (GGGACGGU) and the RBM38-binding motif (UGUGUG) neighbor each other. We hypothesize that RBM45 makes homo-oligomers (42) that bring RBM45-bound ISE2 and ISE3 into proximity such that the A2-2 branch point is preferably more accessible to the D2 site.

In normal cells, RBM45 is localized predominately in the nucleus (43) and plays a role in double-strand break (DSB) repair (44). Cytoplasm-localized RBM45 has been suggested to contribute to neurodegeneration in ALS (45). It is localized within the TDP43-positive cytoplasmic inclusions. This evidence suggests that the aggregation-induced loss of normal RBM45 function might also play an important role in ALS pathogenesis (44). The RNA binding sequence of RBM45 has not been defined. Early experiments indicated that RBM45 protein possesses a binding preference on poly(C) RNA. Later, an RNA motif (“GGGGCC”) was speculated to be bound by and recruit RBM45 to the TDP-43 inclusions in the cytoplasm of neuron cells of ALS patients (45, 62). However, in our study, mutation of the GGGGCC motif in ISE3^26nt^ did not result in a decrease in the binding of RBM45 to this mutant, excluding the possibility that the GGGGCC motif is involved in RBM45 binding. RBM45 binds at high affinity to both ISE2 and ISE3 (*K_D_* values, <34 nM), which share a core octanucleotide (GGGACGGU). This core octanucleotide turned out to be bound by RBM45 at high affinity *in vitro* (*K_D_* = 18 nM). It is possible that those cellular mRNAs that harbor this octanucleotide can sequester RBM45, which plays a role in regulation of mRNA splicing in the nucleus. Under disease conditions, the presence of mislocated RBM45 in the cytoplasm might lead to dysregulation of alternative mRNA splicing or DSB repair in the nucleus. We analyzed 17 available full-length B19V sequences of genotype 1 and found that there are only two sites of the octanucleotide present in the B19V pre-mRNA, namely, ISE2 and ISE3.

In conclusion, the RBM45-binding GGGACGGU octanucleotide is conservative in the pre-mRNA of B19V, which plays an essential role in production of the 11-kDa-encoding mRNA. The identification of the RBM45-binding octanucleotide may have a profound impact on determining which of certain cellular functions or disease conditions are dependent on RBM45.

## MATERIALS AND METHODS

### Ethics statement.

Primary CD34^+^ hematopoietic stem cells were isolated from bone marrow of healthy human donors. We purchased the cells at AllCells (AllCells LLC, Alameda, CA). All of the cell source data were anonymized; therefore, the requirement of an institutional review board (IRB) review was waived.

### Primary cells and cell lines. (i) Primary human CD36^+^ erythroid progenitor cells (EPCs).

Primary human CD36^+^ EPCs were expanded *ex vivo* from CD34^+^ hematopoietic stem cells as previously described (53, 63, 64). Briefly, hematopoietic CD34^+^ stem cells were grown in Wong medium (63, 64) under normoxia conditions (21% O_2_; 5% CO_2_) up to day 4 and frozen in liquid nitrogen. For each experiment, day 4 cells were thawed and cultured for 2 to 3 days in an expansion medium under normoxic conditions. On day 6 or 7, cells were cultured under hypoxic conditions (1% O_2_) for 2 days and subjected to experiments.

### (ii) UT7/Epo-S1 cells.

Human UT7/Epo-S1 cells, representing a megakaryoblastoid cell line that is permissive to B19V infection (65), were cultured under normoxic conditions at 37°C in Dulbecco’s modified Eagle’s medium (DMEM) containing 10% fetal bovine serum and 2 U/ml erythropoietin (Amgen, Thousand Oaks, CA).

### Virus and infection.

A B19V viremic plasma sample (P489) was provided from ViraCor Eurofins Laboratories (Lee’s Summit, MO) and contained 10^12^ viral genome copies (vgc) per ml. CD36^+^ EPCs were infected with B19V at a multiplicity of infection (MOI) of 1,000 vgc/cell (64). The infected cells and media were collected at 2 to 3 days postinfection for Western blotting, Southern blotting, and reverse transcription-quantitative PCR (RT-qPCR) (37).

### Plasmid constructions. (i) ISE3 silent mutation M20 clone (pM20^ISE3-mut7^).

In B19V infectious clone pM20 (containing a dsDNA B19V genome), silent mutations (underlined) (5′-GGA AGG CGA CTG GCA GAT-3′) were introduced into the ISE3-corresponding DNA sequence, as indicated in Fig. 8C.

### (ii) pLKO constructs.

Lentiviral vector pLKO.1 was used with an mCherry reporter (66) to clone shRNA sequences between the AgeI and EcoRI sites. pLKO.1 containing a scramble shRNA sequence was used as a control (64). The following shRNA sequence (corresponding to shRBM45) was obtained from Sigma (St. Louis, MO) for knocking down RBM45: 5′-CCG GCC TTC ATT GAT GAT GGA AGT ACT CGA GTA CTT CCA TCA TCA ATG AAG GTT TTT TG-3′.

### (iii) pLenti-optRBM45^RRM2-HOA-NLS-Flag^ construct.

pLenti vector was used to clone an optimized RBM45 ORF between restriction sites XbaI and SpeI, as described previously (52).

### (iv) Bacterial expression plasmids.

A whole or partial ORF of RBM45 coding sequence was amplified from plasmid pDONR223_RBM45_WT (purchased at Addgene; catalog no. 82892) and cloned into the pET30a (+) vector between the NdeI and XhoI restriction sites. The full-length and truncated RBM45 proteins, as indicated in Fig. 6A, were expressed with a 6×His tag and purified using nickel-nitrilotriacetic acid (Ni-NTA) beads, as described below.

### Transfection.

Two million UT7/Epo-S1 cells were electroporated with 2 μg of SalI-linearized B19V infectious clone pM20 (67) or pM20^ISE3-mut7^ in solution V using an Amaxa Nucleofector system (Lonza, Basel, Switzerland), as described previously (37, 68).

### RNA extraction and RT-qPCR.

Total RNA was extracted from infected or transfected cells using TRIzol reagent (Invitrogen), and cDNA was synthesized using a Moloney murine leukemia virus (M-MLV) kit (Life Technologies, Carlsbad, CA). The multiplex RT-qPCR system for quantification of B19V-specific mRNAs has been described previously (37).

### *In vitro* transcription.

*In vitro* transcription was performed as reported previously (37), with some modifications. Briefly, we used forward primers that had an SP6 RNA polymerase promoter sequence (5′-ATT TAG GTG ACA CTA TAG-3′) at the 5′ end to amplify DNA templates from pM20. The B19V DNA was then transcribed into RNA using SP6 RNA polymerase *in vitro* in the presence of biotin-labeled UTP with biotin-16-UTP (Lucigen, Middleton, WI), following the manufacturer’s instructions. The RNAs were then purified using an RNA Clean and Concentrator-5 kit (Zymo Research, Tustin, CA), following the protocols provided by the manufacturer. Complementary DNAs that were *in vitro* transcribed to complementary RNAs served as negative controls.

### RNA pulldown assay.

The RNA pulldown assay was performed as described previously (37, 69), with some modifications. The RNAs used in the assay were either generated by *in vitro* transcription with SP6 polymerase as described above (11-kDa intron, 11-kDa exon, P1, P2, P3, W1, W2, J1, and J2 and their cRNA sequences; see Fig. 4A to D) or chemically synthesized at Integrated DNA Technologies (Coralville, IA) with biotinylation at the 5′ end (ISE2-WT and ISE2-mut3 [Fig. 1]; ISE3-WT, ISE3^26nt^-mut1 to ISE3^26nt^-mut4, and ISE3^26nt^-com [Fig. 4F and G]; ISE3 and ISE3-mut7 [Fig. 5 and 8, respectively]; and ISE3^8nt^ and ISE3^9nt^ [Fig. 10]). Briefly, 80 μl of streptavidin-conjugated agarose beads (EMD Millipore Corp., Billerica, MA) was washed three times with a wash buffer (50 mM Tris-HCl [pH 7.5], 250 mM NaCl, 1 mM EDTA, 1% NP-40, SigmaFAST EDTA-free protease inhibitor cocktail [Sigma]) and blocked with 3% bovine serum albumin (BSA)–phosphate-buffered saline (PBS) buffer, and 10 μl of 20 μM biotinylated RNA was added. The mixtures were slowly rotated for 3 h at 4°C. The beads were spun down, washed, and resuspended in 500 μl of RNA binding buffer [20 mM Tris-HCl (pH 7.5), 300 mM KCl, 0.2 mM EDTA, 0.5 mM dithiothreitol (DTT), poly(I·C) at 5 μg/ml, proteinase and RNase inhibitors] and 100 μl of nuclear lysate of UT7/Epo-S1 cells or approximately equal amounts of purified proteins. The mixtures were rotated overnight, and the beads were spun down and washed. Bound proteins were suspended in an appropriate volume of 2× Laemmli loading buffer and boiled for 5 to 10 min and were then separated by sodium dodecyl sulfate-polyacrylamide gel electrophoresis (SDS-PAGE), followed by mass spectrometry or immunoblotting.

### Western blotting.

Western blotting was performed as described previously (37, 53, 70, 71). Briefly, the cell lysates or other protein samples were separated by SDS-PAGE. Proteins were transferred onto a nitrocellulose or polyvinylidene difluoride membrane, which was blocked and probed with primary and secondary antibodies sequentially. Signals were visualized by enhanced chemiluminescence, and images were developed under a Fuji LAS3000 imaging system.

### Mass spectrometry.

Protein bands differentially detected in SDS-PAGE that corresponded to the samples pulled down by ISE2-WT RNA but not those pulled down by ISE2-mut3 RNA were excised and subjected to protein identification using microcapillary reverse high-performance liquid chromatography–nanoscale electrospray tandem mass spectrometry (μLC-MS/MS) on a Finnigan LCQ quadrupole ion trap mass spectrometer at the Taplin Biological Mass Spectrometry Facility, Harvard University (Cambridge, MA).

### Immunofluorescence.

B19V-infected CD36^+^ EPCs were collected spun onto slides at 1,800 rpm for 5 min using a Cytospin centrifuge. After being fixed and permeabilized in 0.5% Triton X-100, the slides were then blocked and probed with primary antibodies, including rabbit anti-RBM45 antibodies (Abclonal; catalog no. A13843) and mouse anti-B19V capsid antibodies (Millipore; catalog no. MAB8293), followed by incubation with Alexa Fluor 594-conjugated anti-rabbit IgG and fluorescein isothiocyanate (FITC)-conjugated anti-mouse IgG secondary antibodies. The slides were visualized under a 63× oil lens objective with a Leica TCS SPE confocal microscope. DAPI (4′,6-diamidino-2-phenylindole) was used to stain the nucleus.

### Proximity ligation assay.

Proximity ligation assay (PLA) was performed using a Duolink PLA kit (Sigma) according to the manufacturer’s instructions (66). Briefly, infected CD36^+^ EPCs were fixed, permeabilized as described above, and blocked with Duolink blocking buffer for 30 min. The cells were then incubated with primary antibodies (rabbit anti-RBM45 and mouse anti-B19V capsid antibodies) for 1 h. The PLA probe incubation, ligation, and amplification processes were performed according to the manufacturer’s instructions. Finally, the cells were washed and mounted with Duolink *in situ* mounting medium with DAPI and visualized under the 63× oil lens objective with a Leica TCS SPE confocal microscope.

### BrdU incorporation assay and flow cytometry.

For cell cycle analysis of CD36^+^ EPCs, a bromodeoxyuridine (BrdU) incorporation assay was performed to determine the percentage of the cell population in S phase, followed by flow cytometry analysis, as reported previously (70).

### Northern blotting.

Northern blot analysis was performed as previously described (72, 73). In brief, total RNA was extracted from pM20-transfected or pM20^ISE3-mut7^-transfected UT7/Epo-S1 cells by the use of TRIzol reagent (Invitrogen). A 5-μg volume of the total RNA was separated on a denatured 1.4% agarose gel and visualized using ethidium bromide (EB) staining. The stained 28S ribosome RNA band served as a loading control. For the detection of 11-kDa mRNA, the blot was hybridized with a [^32^P]dCTP-labeled DNA *cap* probe (nt 4619 to 5147), which was amplified from pM20 by PCR. Hybridization signals were captured by the use of a storage phosphor screen and visualized on a Typhoon FLA 9000 biomolecular imager (GE Healthcare). Quantification of the bands was carried out using ImageQuant TL 8.1 software (GE Healthcare).

### Southern blotting.

Hirt DNA (lower-molecular-weight DNA) was extracted from either B19V dsDNA genome (pM20)-transfected UT7/Epo-S1 cells or B19V-infected CD36^+^ EPCs, as reported previously (37, 68). B19V RF DNA M20 excised from SalI-digested pM20 was used as a probe. The blots were reprobed for mitochondrial DNA (Mito-DNA) using a specific probe (74). Hybridization signals were captured by the use of the Typhoon FLA 9000 biomolecular imager, and quantification of the bands was carried out using ImageQuant TL.

### Biolayer interferometry assay.

Biolayer interferometry (BLI) was performed as reported (37), with minor modifications. Briefly, biotinylated RNA was mounted onto streptavidin biosensors (catalog no. 18-5019; Forte Bio Inc., Fremont, CA), equilibrated with RNA binding buffer (20 mM Tris-HCl [pH 7.4], 80 mM NaCl), and then dipped into the binding buffer containing purified BM45 at different concentrations. Binding parameters *K*_ass_ (association rate constant) and *K*_diss_ (dissociation rate constant) were generated from Blitz Pro software. *K_D_* (equilibrium dissociation constant) values were calculated by dividing *K*_ass_ with *K*_diss_.

### Lentivirus production and transduction.

Lentiviruses were produced according to instructions provided by Addgene (https://www.addgene.org/protocols/lentivirus-production/) and were concentrated as described previously (64). UT7/Epo-S1 cells or CD36^+^ EPCs were transduced with the lentiviral vector at a multiplicity of infection (MOI) of approximately 5 transduction units per cell, as described previously (64).

### Protein purification.

The cloned pET30a (+) plasmids were transformed in Escherichia coli BL21(DE3)/pLysS cells (Promega, Madison, WI). The transformation reaction mixtures were inoculated in 5 ml of 2× YT medium containing 25 μg/ml of kanamycin and cultured overnight at 37°C in an incubator shaker at 250 rpm. A 5-ml volume of each culture was used to inoculate 1 liter of 2× YT medium containing 25 μg/ml of kanamycin. When the optical density at 600 nm (OD_600_) of bacterial culture reached approximately 0.4 to 0.6, IPTG (isopropyl-β-d-thiogalactopyranoside; final concentration of 0.5 mM) was added to the media and the culture was grown for another 2 to 3 h at 37°C or overnight at 16°C. His-tagged recombinant proteins were purified as described previously (37). The purified proteins were stored at −80°C for use.

### Antibodies used.

The following antibodies were used: rabbit anti-RBM45 (ab123912 [for Western blotting]) and rabbit anti-DHX37 (ab70778) from Abcam (Cambridge, MA); rabbit anti-RBM45 (A13843 [for immunofluorescence]), rabbit anti-MCM7 (A1138), rabbit anti-hnRNP D (A15679), rabbit anti-PURA (A9296), and rabbit anti-HuR (A19622) from Abclonal (Woburn, MA); rabbit anti-DHX9 (A300-855A), rabbit anti-SF3B1 (A300-996A), rabbit anti-KHSRP (A302-021A), rabbit anti-DDX21 (A300-629A), rabbit anti-LARP7 (A300-724A), rabbit anti-NOP56 (A302-720A), rabbit anti-hnRNP H1 (A300-511A), and rabbit anti-DDX21 (A300-629A) from Bethyl Laboratories (Montgomery, TX); rabbit anti-FUBP1 (GTX1045879), rabbit anti-hnRNP R (GTX106526), rabbit anti-hnRNP A2B1 (GTX127928), and rabbit anti-SF2 (GTX114918) from GeneTex (Irvine, CA); rabbit anti-hnRNP G (AP21472C) from Abgent (San Diego, CA); rabbit anti-hnRNP A3 (25142-1-AP) from Proteintech Group Inc. (Chicago, IL); mouse anti-β-actin (A5441) from MilliporeSigma (St. Louis, MO); mouse anti-B19V capsid (MAB8292) from Millipore (Billerica, MA); rabbit anti-GST (2625T) from Cell Signaling Technology (Danvers, MA); and mouse anti-Flag (2001-301-B13) from Rockland (Limerick, PA). Rat anti-11-kDa protein and anti-NS1 antibodies were produced in-house, as described previously (29).

The secondary antibodies used were horseradish peroxidase (HRP)-conjugated anti-mouse IgG (A4416) and HRP-conjugated anti-rat IgG (A9037) from MilliporeSigma (St. Louis, MO) and HRP-conjugated anti-rabbit IgG (sc-2357) from Santa Cruz (Dallas, TX).

### Statistical analysis.

Statistical analysis was done by using GraphPad Prism version 8.0. Error bars represent means and standard deviations (SD), and statistical significance *P* values were determined by using Student's *t* test. Statistical significance is indicated (****, *P* < 0.0001; ***, *P* < 0.001; **, *P* < 0.01; *, *P* < 0.05; n.s, no statistical significance). For cell cycle analysis, the percentage of cells in S phase was determined from the results of at least three independent experiments. For RT-qPCR analysis, quantified viral mRNA levels were normalized to the level of β-actin mRNA, and the value representing mRNAs extracted from control cells was arbitrarily set at 1, with at least three independent experiments performed.
