# Supplementary figures and images for: RNA Binding Motif Protein RBM45 Regulates Expression of the 11-Kilodalton Protein of Parvovirus B19 through Binding to Novel Intron Splicing Enhancers
Source: mBio. 2020 Mar 10;11(2):e00192-20. doi: 10.1128/mBio.00192-20 (PMC7064759; doi:10.1128/mBio.00192-20)

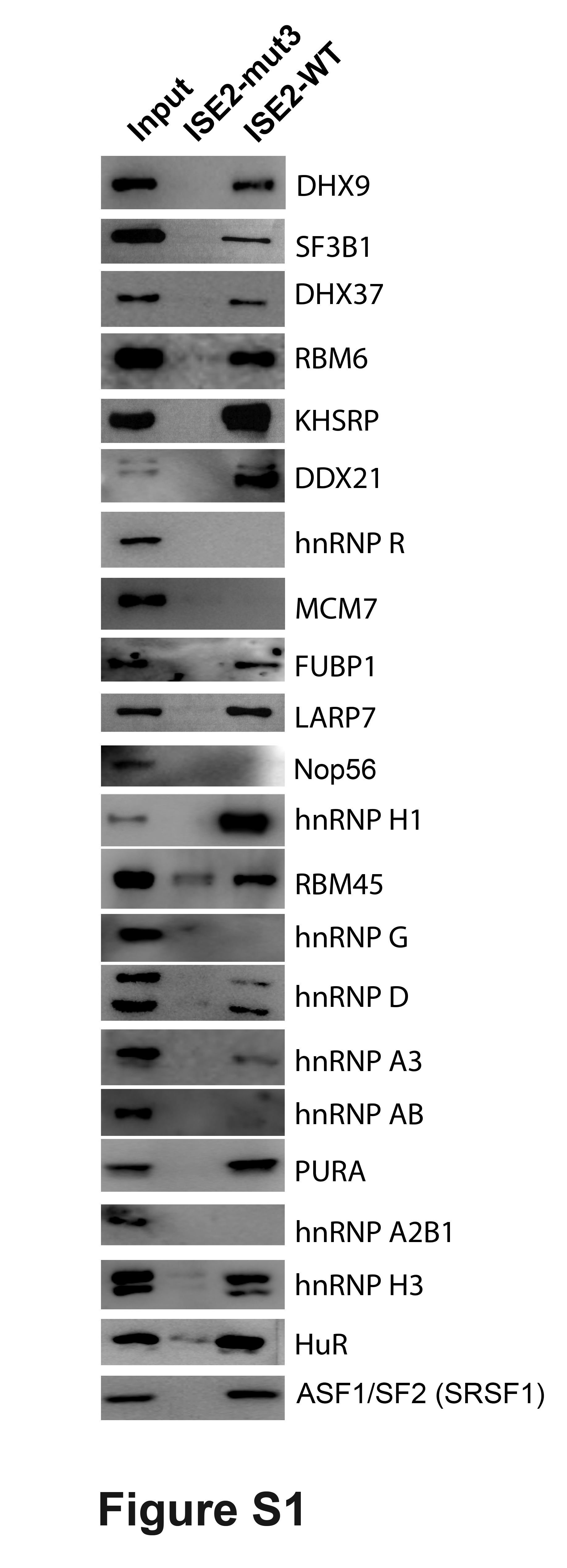

Supplement: FIG S1 [file mBio.00192-20-sf001.tif]

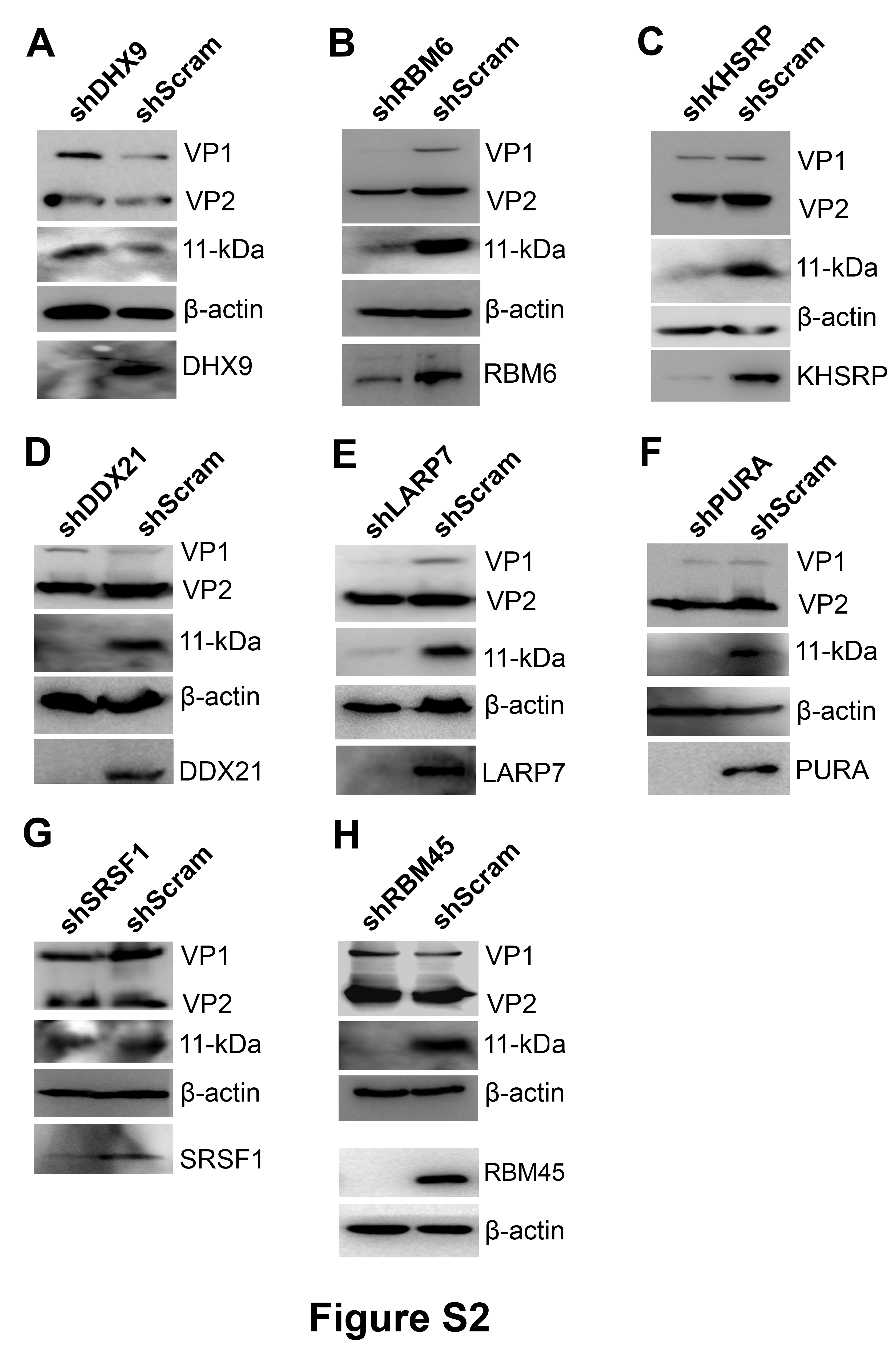

Supplement: FIG S2 [file mBio.00192-20-sf002.tif]
