# Supplementary material for: RNA Binding Motif Protein RBM45 Regulates Expression of the 11-Kilodalton Protein of Parvovirus B19 through Binding to Novel Intron Splicing Enhancers
Source: mBio. 2020 Mar 10;11(2):e00192-20. doi: 10.1128/mBio.00192-20 (PMC7064759; doi:10.1128/mBio.00192-20)
Supplement: TABLE S1 [file mBio.00192-20-st001.docx]

| **Sample No.** | **Total**  **reads** | **Unique**  **reads** | **Accession no.** | **Protein** | **Functions** | **ISE2 pulldown/**  **Decrease in 11-kDa expression** |
| --- | --- | --- | --- | --- | --- | --- |
| **B1** | 607 | 69 | Q08211 | **DHX9** | dsRNA/RNA-DNA helicase | +/ ̶ |
|  | 95 | 44 | O75533 | SF3B1* | Splicing/U2 snRNP | +/ND |
|  | 33 | 30 | P53651 | *COPA* | ER transport | ND |
|  | 37 | 29 | Q8IY37 | DHX37* | RNA helicase | +/ND |
|  | 31 | 25 | P78332 | **RBM6** | RNA binding | +/+ |
|  | 24 | 23 | P35251 | RFC1 | DNA replication | ND |
| **B2** | 1728 | 68 | Q92945 | **KHSRP** | RNA stability/Splicing | +/+ |
|  | 164 | 43 | P13010 | *XRCC5* | DNA repair | ND |
|  | 27 | 20 | Q9NR30 | **DDX21** | RNA helicase | +/+ |
| **B3** | 1330 | 58 | Q92945 | ***KHSRP*** |  |  |
|  | 53 | 34 | Q13616 | CUL1 | protein ubiquitination | ND |
|  | 35 | 28 | P13010 | ***XRCC5*** |  |  |
|  | 216 | 26 | O43390 | hnRNP R | Pre-mRNA splicing | ̶ |
|  | 32 | 26 | P33993 | MCM7 | DNA replication | ̶ |
|  | 24 | 21 | Q02809 | Pol δ1 | DNA replication | ND |
| **B4** | 1518 | 58 | Q96AE4 | FUBP1* | DNA/RNA binding | +/ND |
|  | 46 | 31 | Q4G0J3 | **LARP7** | Transcription/7SK snRNP | +/+ |
| **B5** | 1475 | 58 | Q96QE4 | ***FUBP1*** |  |  |
|  | 41 | 35 | O00567 | Nop56 | snoRNP/splicesosome | ̶ |
| **B6** | 724 | 35 | P31943 | hnRNP H1 | RNA processing | + |
|  | 82 | 25 | Q13885 | *TUBB2A* | microtubules | ND |
|  | 114 | 23 | Q71U36 | *TUBA1A* | microtubules | ND |
|  | 40 | 21 | Q8IUH3 | **RBM45** | RNA binding | +/+ |
| **B7** | 185 | 31 | P38159 | hnRNP G | RNA binding/splicing | ̶ |
|  | 53 | 24 | Q12905 | *ILF2* | transcription factor | ND |
|  | 77 | 22 | Q14103 | hnRNP D | RNA processing | + |
|  | 21 | 20 | P60842 | *eIF4A1* | mRNA translation | ND |
| **B8** | 410 | 31 | Q14103 | ***hnRNP D*** |  |  |
|  | 351 | 28 | P51991 | hnRNP A3 | RNA processing | + |
|  | 200 | 22 | Q99729 | hnRNP AB | RNA processing | ̶ |
|  | 252 | 20 | Q00577 | **PURA** | transcription activator | +/+ |
|  | 80 | 20 | P22626 | hnRNP A2B1 | RNA processing | ̶ |
| **B9** | 152 | 27 | P22626 | ***hnRNPA 2B1*** |  |  |
|  | 30 | 27 | P82650 | *MRPS22* | Mitochondrial translation | ND |
|  | 86 | 23 | Q99729 | ***hnRNP AB*** |  |  |
|  | 62 | 22 | P31942 | hnRNPH3 | RNA processing | + |
|  | 24 | 20 | P51991 | ***hnRNP A3*** |  |  |
| **B10** | 168 | 32 | P22626 | ***hnRNP A2B1*** |  |  |
|  | 89 | 20 | P31942 | ***hnRNP H3*** |  |  |
| **B11** | 18 | 18 | Q15717 | HuR | RNA-binding | +/ND |
|  | 69 | 17 | P22626 | ***hnRNP A2B1*** |  |  |
|  | 19 | 17 | Q07955 | **ASF/SF2** | RNA processing | +/ ̶ |

**Table S1. Mass spectrometry-identified 32 proteins with unique reads of ≥20 or the top 3 in the band.**

Notes: 10 proteins, which are underlined, do not have a function in RNA biogenesis as analyzed by UniProt (<https://www.uniprot.org/>) and were not tested for RNA binding. All shRNP proteins were not tested by knockdown as they function as negative regulators. SF3b1 is essential for cell growth, and DHX37 and FUBP1 were not successfully knocked down in UT7/Epo-S1 cells, which are marked with a star (*). ND: not determined.
