# Supplementary material for: RNA Binding Motif Protein RBM45 Regulates Expression of the 11-Kilodalton Protein of Parvovirus B19 through Binding to Novel Intron Splicing Enhancers
Source: mBio. 2020 Mar 10;11(2):e00192-20. doi: 10.1128/mBio.00192-20 (PMC7064759; doi:10.1128/mBio.00192-20)
Supplement: TABLE S2 [file mBio.00192-20-st002.docx]

| **Name of shRNA** | **Sequence (5’-3’)** |
| --- | --- |
| **shDHX9-4** | TAA ATT ATG ATC TTG TTC CAT |
| **shRBM6-2** | CAA ATG TAG AGG AGC ATT CTT |
| **shKHSRP-3** | CGC CTA CTA CTC ACA CTA CTA |
| **shDDX21-3** | CCC ATA TCT GAA GAA ACT ATT |
| **shLARP7-9** | CAG AAT GCC ATG CTA GAT TTA |
| **shPURA-1** | GAG CCG CCT TAC TCT CTC CAT |
| **shSRSF1-2** | ACT TAC CTC CAG ACA TCC GAA |

**Table S2 shRNA targeting sequences used to knock done genes listed in Fig. S2.**
